# Intermediates involved in serotonin oxidation catalyzed by Cu bound Aβ peptides†

**DOI:** 10.1039/d0sc06258h

**Published:** 2020-12-22

**Authors:** Arnab Kumar Nath, Arnab Ghatak, Abhishek Dey, Somdatta Ghosh Dey

**Affiliations:** Indian Association for the Cultivation of Science 2A & 2B, Raja S. C. Mullick Road, Jadavpur Kolkata 700032 India icsgd@iacs.res.in

## Abstract

The degradation of neurotransmitters is a hallmark feature of Alzheimer's disease (AD). Copper bound Aβ peptides, invoked to be involved in the pathology of AD, are found to catalyze the oxidation of serotonin (5-HT) by H_2_O_2_. A combination of EPR and resonance Raman spectroscopy reveals the formation of a Cu(ii)–OOH species and a dimeric, EPR silent, Cu_2_O_2_ bis-μ-oxo species under the reaction conditions. The Cu(ii)–OOH species, which can be selectively formed in the presence of excess H_2_O_2_, is the reactive intermediate responsible for 5-HT oxidation. H_2_O_2_ produced by the reaction of O_2_ with reduced Cu(i)–Aβ species can also oxidize 5-HT. Both these pathways are physiologically relevant and may be involved in the observed decay of neurotransmitters as observed in AD patients.

## Abstract 2

The mononuclear copper hydroperoxo species (Cu(ii)–OOH) of Cu–Aβ is the active oxidant responsible for serotonin oxidation by Cu–Aβ in the presence of physiologically relevant oxidants like O_2_ and H_2_O_2_, which can potentially cause oxidative degradation of neurotransmitters, a marker of Alzheimer's disease.

## Graphical abstract

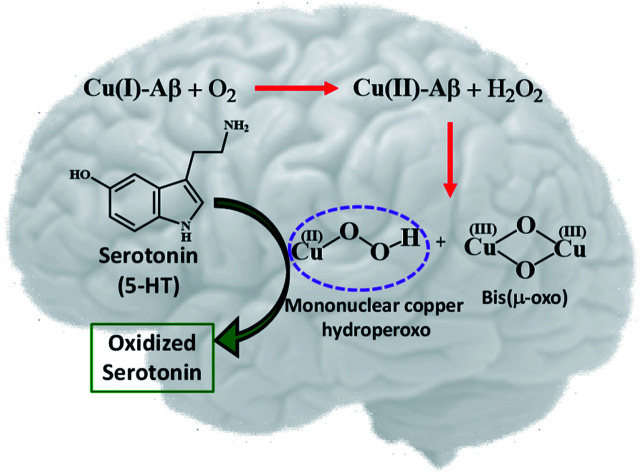

## Introduction

Alzheimer's disease (AD) is a terminal neurodegenerative disease which is characterized by the deposition of insoluble plaques in the hippocampus of an AD affected brain.^1,2^ It is clinically characterized by progressive dementia.^3^ The exact cause for this disease is still unclear. Over the past two decades extensive research has focused on determining the actual cause of this disease, resulting in important hypotheses relating to the pathology of AD. Among them, one of the more accepted hypothesis is the “amyloid hypothesis”.^4–6^ The amyloid β-peptide is the main constituent of this hypothesis and is found in high proportions in the plaques that are diagnostic of AD.^5,6^ Moreover transition metals like Cu(ii) and Zn(ii) and cofactors like heme are found in the plaques of an AD brain.^7–9^ Apart from the ability of these bivalent metals to aggregate the monomeric Aβ peptides, redox active metals like Cu and Fe are proposed to cause oxidative stress resulting in damage to the neuronal cell membrane making it “leaky”.^7,9^ The oxidative damage is proposed to be an upstream event which eventually leads to the formation of insoluble plaques which is a hallmark of the disease.^7,10^ The oxidative stress can be due to hydrogen peroxide (H_2_O_2_), which, apart from its natural availability, can also be produced by the reaction of the reduced redox active metals bound Aβ peptides with O_2_.^9,11,12^

**Scheme 1:**
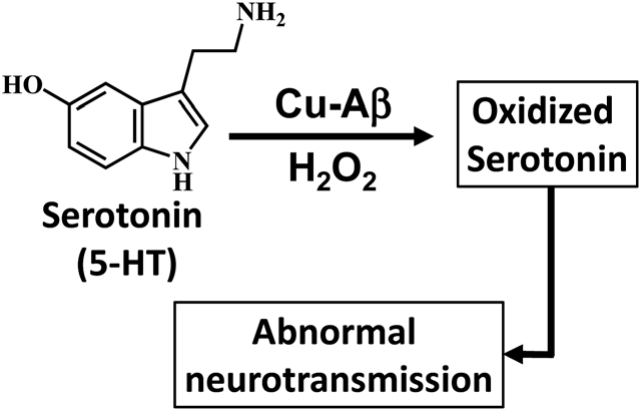
Oxidation of the neurotransmitter serotonin (5-HT) by Cu–Aβ and H_2_O_2_.

The recent investigation of Cu bound Aβ peptides and their site-directed mutants using a combination of spectroscopic techniques and theoretical calculations has revealed a Cu active site coordinated to histidine residues and exchangeable ligands.^13–18^ The Cu–Aβ complexes exhibit peroxidase activity in the presence of H_2_O_2_.^12^ Presently several naturally occurring copper containing metalloenzymes are known which can oxidize/hydroxylate organic compounds in the presence of H_2_O_2_. Enzymes like amine oxidase participate in the breakdown of amines to produce an aldehyde and ammonia.^19–21^ Alternatively, copper enzymes like dopamine β-hydroxylase (DβM) are involved in the synthesis of a small-molecule neurotransmitter by catalyzing the hydroxylation of dopamine to convert it to norepinephrine.^19,22–24^ Note that the Cu active sites of these above-mentioned proteins also have histidine and water derived exchangeable ligands in their active sites similar to that of Cu–Aβ. Now oxidative degradation of neurotransmitters like serotonin generating neurotoxins like tryptamine-4,5-dione is a hallmark of AD, which can lead to impaired neuronal signaling.^25–33^ This raises the possibility of Cu bound Aβ reacting with H_2_O_2_ and catalyzing the degradation of neurotransmitters (Scheme 1). Previously Ming *et al.* have kinetically shown the catalytic hydroxylation/oxidation of substrates like dopamine, catechol derivatives, phenol and serotonin and proposed a side-on μ-peroxo dicopper(ii) intermediate as the active species, though no spectroscopic proof was provided.^34–37^ Since the oxidation of neurotransmitters by Cu–Aβ in the presence of H_2_O_2_ is a likely physiological process occurring in the human brain, the identification and characterization of the reactive intermediates involved in this chemical oxidation process are crucial for the understanding of this disease.

In this manuscript, we experimentally determine the reaction intermediates involved in serotonin oxidation as well as in the peroxidase activity pathway of Cu(ii) bound Aβ peptides in the presence of H_2_O_2_. Spectroscopic evidence reveals that two intermediates, Cu_2_O_2_-bis-μ-oxo and Cu(ii)–OOH, are formed in the reaction of Cu–Aβ and H_2_O_2_.

## Results and discussion

### Neurotransmitter serotonin (5-HT) oxidation by Cu–Aβ with H_2_O_2_

**Fig. 1:**
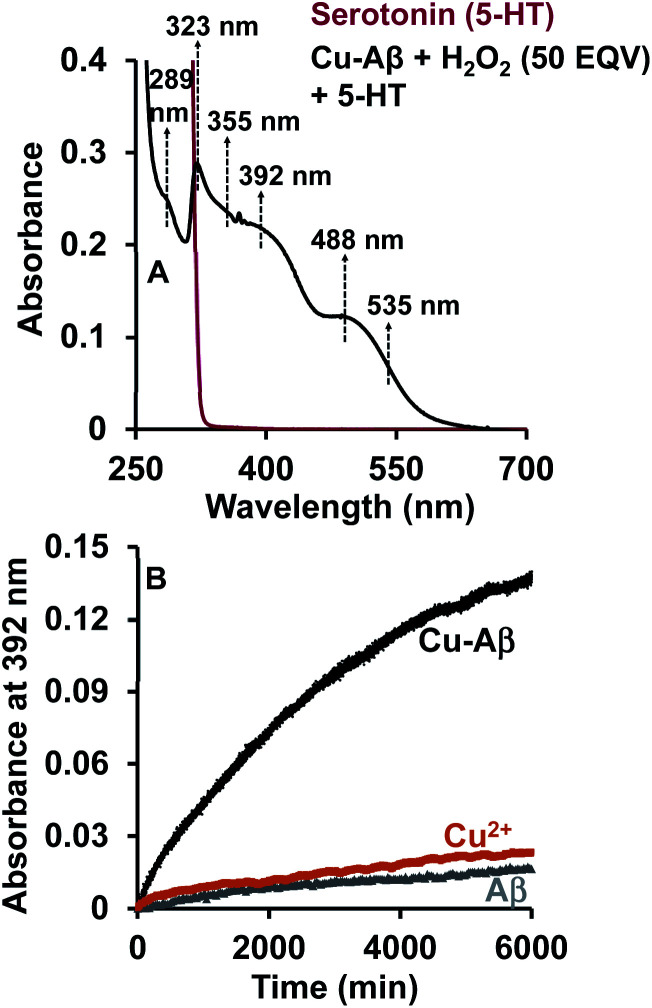
(A) Absorption spectrum of serotonin (5-HT), red; 5-HT + Cu–Aβ + 50 eq. H_2_O_2_, black; (B) kinetics of serotonin oxidation monitored at 392 nm; 5-HT + Cu–Aβ + 50 eq. H_2_O_2_, black; 5-HT + CuSO_4_ + 50 eq. H_2_O_2_, brown; 5-HT + Aβ + 50 eq. H_2_O_2_, grey; in 100 mM HEPES buffer at pH 7. [Cu–Aβ = 0.055 mM, H_2_O_2_ = 2.77 mM and 5-HT = 2.77 mM].

The reaction of the neurotransmitter serotonin (5-HT) with Cu–Aβ in the presence of H_2_O_2_ produces absorption peaks at 289, 323, 355, 392, 488 and 535 nm (Fig. 1A and S1†). These peaks are indicative of serotonin oxidation. Note that, as Cu–Aβ (1–16) and Cu–Aβ (1–40) produce similar serotonin oxidized products, all the other experiments have been performed with Cu–Aβ (1–16). The oxidized products of serotonin are further characterized using HPLC as previously reported (Fig. S2 and Scheme S1†).^38,39^ The products are found to be tryptamine-4,5-dione (T-4,5-D), 5-hydroxy-3-ethylamino-2-oxindole (5-HEO) and 3,3′-bis(2-aminoethyl)-5-hydroxy-[3,7′-bi-1*H*-indole]-2,4′,5′(3*H*)-trione, which is the aerially oxidized dimer of T-4,5-D and 5-HEO. The pseudo 1^st^ order rate for this reaction is found to be (4 ± 0.2) × 10^−4^ s^−1^ (Fig. 1B and S3†). Note that the slow kinetics of this oxidation matches the slow onset and progression of this disease. Control experiments without Cu–Aβ, *i.e.* H_2_O_2_ or Cu(ii) (Fenton reaction conditions) alone, show barely any oxidation of 5-HT (Fig. 1B).

**Scheme 2:**
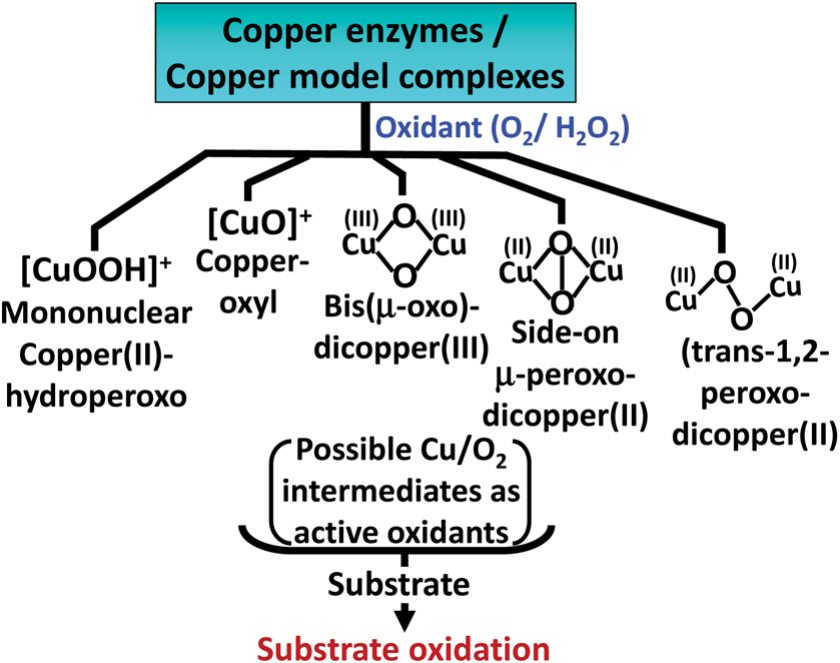
Possible reactive Cu/O_2_ intermediates for substrate oxidation by different copper enzymes and copper model complexes in the presence of oxidants (O_2_ or H_2_O_2_).^19,40–42^

The oxidation of 5-HT by H_2_O_2_ catalyzed by Cu(ii)–Aβ can proceed *via* several reactive intermediates similar to those which have been observed in different Cu enzymatic active sites and their synthetic analogues^19,40–43^ (Scheme 2). Hence the reaction of Cu–Aβ with H_2_O_2_ is monitored with the aim of trapping and characterizing the active oxidant responsible for oxidizing substrates like serotonin and other neurotransmitters.

### Characterization of Cu/O_2_ intermediates formed in the reaction of Cu–Aβ with H_2_O_2_

#### Absorption spectroscopy

**Fig. 2:**
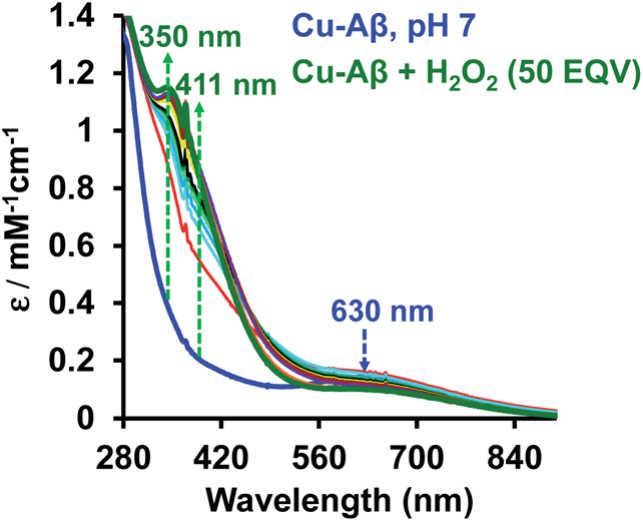
Absorption spectrum of Cu–Aβ, blue; Cu–Aβ with 50 eq. of H_2_O_2_ at different times, green being the final spectrum after 120 minutes; data are collected in 100 mM HEPES buffer at pH 7. The arrows indicate the direction of the spectral changes.

At physiological pH, Cu–Aβ shows a broad ligand field band at 630 nm. The addition of H_2_O_2_ to Cu–Aβ produces charge transfer (CT) bands at 350 nm (*ε* = 1200 M^−1^ cm^−1^) and 411 nm (*ε* = 850 M^−1^ cm^−1^) (Fig. 2). The energies of these CT bands are characteristic of peroxide 
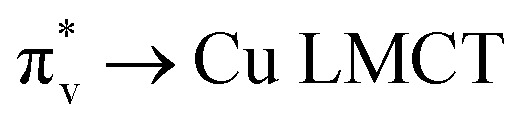
. CT bands in these regions hint at the plausible formation of either a side-on μ-peroxo-dicopper(ii) complex or a bis(μ-oxo)dicopper(iii) complex as depicted in Scheme 2.^41,42,44–46^ Previously Ming and coworkers proposed the formation of a side-on μ-peroxo dicopper(ii) intermediate.^34,36^

#### EPR spectroscopy

**Fig. 3:**
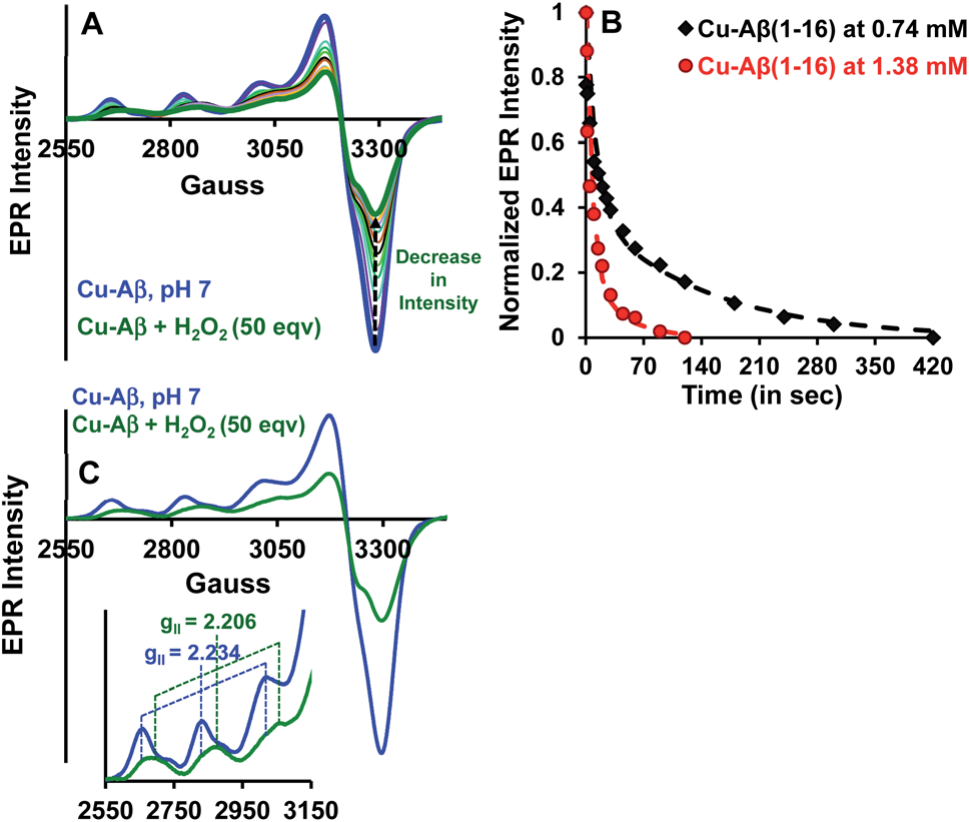
(A) EPR spectra of Cu–Aβ, blue; Cu–Aβ with 50 eq. H_2_O_2_ at different times, green being the final spectrum after 120 minutes; (B) kinetics of the loss of the EPR signal for 50 eq. H_2_O_2_ with 0.74 mM Cu–Aβ, black and 1.38 mM Cu–Aβ, red; (C) EPR spectra of Cu–Aβ, blue; Cu–Aβ with 50 eq. H_2_O_2_, green, inset: hyperfine overlay of the data; data are obtained in 100 mM HEPES buffer at pH 7 and 77 K.

Both side-on peroxo and bis(μ-oxo) complexes are diamagnetic (*S* = 0) and hence are expected to be EPR silent.^44,47,48^ The native Cu–Aβ at pH 7 shows a characteristic (*S* = 1/2) axial EPR signal (predominantly component I with a trace of component II) (Fig. 3A and C).^13,16^ When H_2_O_2_ is added to Cu–Aβ, ∼40% loss of the EPR signal is observed relative to that of the starting Cu–Aβ signal (Fig. 3A and S4†). A loss in EPR signal intensity can be indicative of the formation of a diamagnetic side-on μ-peroxo- dicopper(ii) or a bis(μ-oxo)dicopper(iii) species. The formation of a dimeric species from mononuclear Cu–Aβ should result from the dimerization of two monomeric Cu species. Accordingly, as the Cu–Aβ concentration is doubled, the rate of spin loss of the Cu–Aβ EPR signal is doubled (Fig. 3B). This indicates that the side-on μ-peroxo-dicopper(ii) or bis(μ-oxo)dicopper(iii) bridging is intermolecular resulting in the formation of a diamagnetic species. Note that in this reaction, the EPR signal of the reaction mixture of Cu–Aβ and H_2_O_2_ did not disappear completely (Fig. 3A). Thus, the remaining spin on Cu–Aβ after the reaction is likely due to the presence of unreacted Cu–Aβ or the formation of another paramagnetic species. The EPR parameters of the residual paramagnetic species (*S* = 1/2, *g*_II_ = 2.206) (Fig. 3A, C and Table 1) indicate the formation of a distinct species and is not consistent with residual unreacted Cu–Aβ. The new diamagnetic and paramagnetic species observed in absorption and EPR spectroscopy have characteristic vibrations and have been further probed using resonance Raman spectroscopy.

**Table 1:** EPR parameters of Cu–Aβ and a Cu–Aβ + 50 eq. H_2_O_2_ mixture at pH 7

Complex	*A*_ll_	*g*_ll_
Cu–Aβ	173	2.234
Cu–Aβ + 50 eq. H_2_O_2_	166	2.206

#### Resonance Raman spectroscopy

**Fig. 4:**
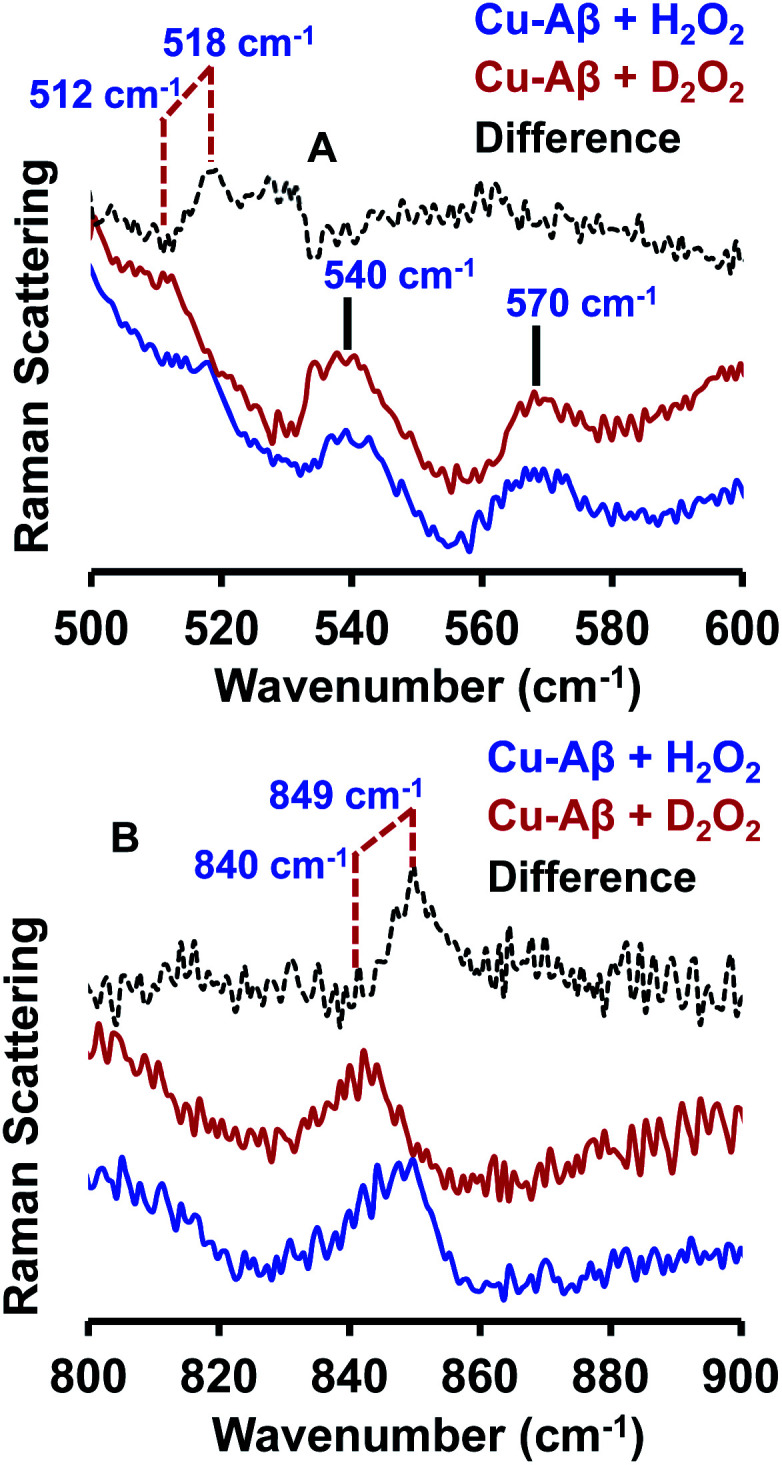
Resonance Raman spectra of (A) Cu–Aβ + 50 eq. H_2_O_2_, blue and Cu–Aβ + 50 eq. D_2_O_2_ in, red; difference spectrum of H_2_O_2_ data from D_2_O_2_ data, dashed black; lower energy region; (B) Cu–Aβ + 50 eq. H_2_O_2_, blue and Cu–Aβ + 50 eq. D_2_O_2_ in, red; difference spectrum of H_2_O_2_ data from D_2_O_2_ data, dashed black; higher energy region. Data were obtained with an excitation wavelength of 415.4 nm (10 mW at the sample) at 77 K (full spectra in Fig. S5†).

**Scheme 3:**
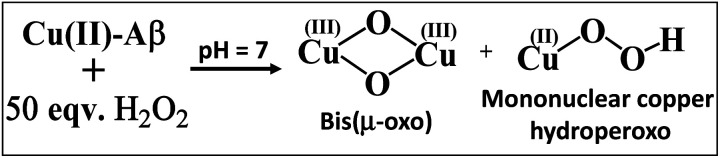
Reaction of Cu–Aβ with 50 eq. H_2_O_2_ at pH 7.

The resonance Raman spectra of the frozen sample of the reaction mixture of Cu–Aβ and H_2_O_2_ is obtained using an excitation wavelength of 415.4 nm at 77 K. The reaction mixture of Cu–Aβ and H_2_O_2_ exhibits resonance Raman bands at 518, 540, 570 and 849 cm^−1^ (Fig. 4). Bands in the range of 500–600 cm^−1^ are characteristic of Cu–O vibrations of either a bis(μ-oxo)copper(iii) core or a Cu(ii)–OOH species, whereas a band at around 849 cm^−1^ is characteristic of O–O vibration of a Cu(ii)–OOH species.^40–42,49–51^ Note that the characteristic Cu–O and O–O vibrations of the side-on μ-peroxo-dicopper(ii) complex are usually observed at ∼580 and ∼750 cm^−1^ respectively.^41,44^ These assignments can be verified by isotopic substitution of the oxygen or proton of H_2_O_2_. The reaction mixture of Cu–Aβ and H_2_O_2_ in a deuterated medium shows a shift of the bands from 849 and 518 cm^−1^ to 840 and 512 cm^−1^ respectively (Fig. 4B). However, the bands at 540 and 570 cm^−1^ remain unperturbed (Fig. 4A). This H/D shift of the 849 and 518 cm^−1^ bands is comparable with those reported for several Cu–OOH and Fe–OOH species.^51–57^ Therefore the 849 cm^−1^ band can be assigned to the O–O vibration and the 518 cm^−1^ band can be assigned to the Cu–O vibration of a Cu(ii)–OOH species of Cu–Aβ, and represents the new paramagnetic species observed in the EPR spectrum (Fig. 3 and Table 1) when H_2_O_2_ is added to Cu–Aβ. The two bands at 540 and 570 cm^−1^ which do not show any deuterium shift arise from a Fermi resonance (Fig. 4A) and are characteristic Cu–O vibrations of a diamond core bis(μ-oxo) species.^45,48,49^ Hence, the doublet in the resonance Raman spectra, CT bands at 350 nm and 411 nm and EPR inactivity (*S* = 0) are all consistent with the formation of a bis(μ-oxo)dicopper(iii) species. Thus, Cu–Aβ reacts with H_2_O_2_ to generate two different Cu/O_2_ species; a bis(μ-oxo)dicopper(iii) species and a mononuclear Cu(ii)–OOH species as shown in Scheme 3. Note that the *trans*-1,2-peroxo dicooper(II) (Cu–OOCu) could be another possibility but is excluded since the UV-Vis spectrum of Cu–Aβ + H_2_O_2_ does not possess any intense band in the range between 480 and 550 nm, which is characteristic of a *trans*-1,2-peroxo dicooper(II) species.^40,41,58,59^ Moreover, it has a characteristic O–O vibration in between 800 and 830 cm^−1^ which is very low as compared to a mononuclear Cu(ii)–OOH species, which shows O–O stretch ranging from 830 to 880 cm^−1^.^40,41,51,52^ Most importantly the *trans*-1,2-peroxo species does not show any H/D shift, which is seen in a mononuclear Cu(ii)–OOH species.^40,41,51–53,59^

#### Active oxidant

**Fig. 5:**
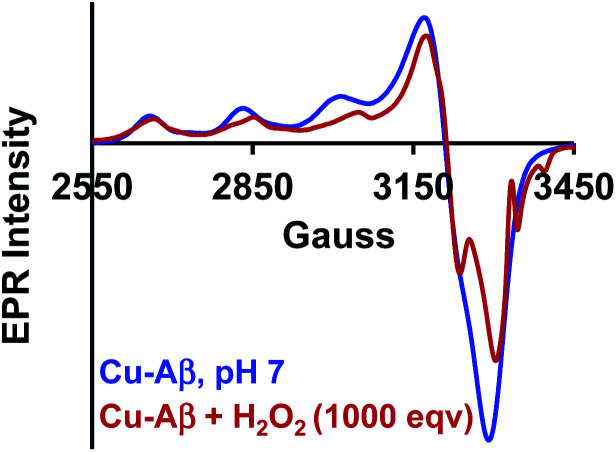
EPR spectra of Cu–Aβ, blue; for the reaction of Cu–Aβ with 1000 eq. H_2_O_2_, red; in 100 mM HEPES buffer at pH 7, at 77 K.

**Scheme 4:**
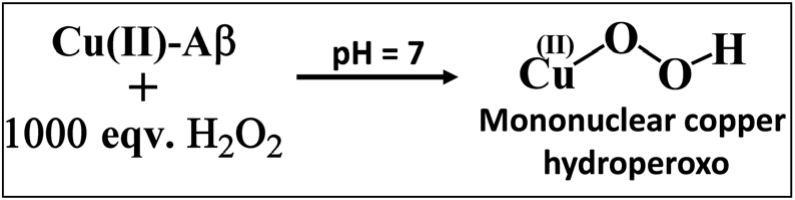
Reaction of Cu–Aβ with 1000 eq. H_2_O_2_ at pH 7.

The Cu_2_O_2_ bis(μ-oxo)dicopper(iii) dimer can be formed by a reaction between two equivalents of Cu–Aβ and one equivalent of H_2_O_2_. Alternatively, Cu–Aβ can react with H_2_O_2_ to form Cu(ii)–OOH which then dimerizes to Cu_2_O_2_ bis(μ-oxo)dicopper(iii) in the presence of another Cu–Aβ. We find that when Cu–Aβ reacts with an increased amount of H_2_O_2_ (1000 equivalents) keeping everything else the same, the EPR spectrum shows that there is no loss of spin during this reaction (Fig. 5) and parallel rR data do not reveal the formation of the dimeric Cu_2_O_2_ bis(μ-oxo)dicopper(iii) species. Although the O–O vibration of the Cu(ii)–OOH species is masked by the O–O vibration of free H_2_O_2_ in solution at 890 cm^−1^, the Cu–O vibration and its shift in D_2_O are clearly observed (Fig. S6†). Thus under these reaction conditions, Cu(ii)–OOH is formed exclusively (Scheme 4). Interestingly, the oxidation of 5-HT continues to occur under these conditions (Fig. S7, S8 and Table S1†). Note that the use of 1000 eq. H_2_O_2_ is not physiologically relevant and the purpose of the experiment is to avoid dimerization and to generate mononuclear Cu(ii)–OOH exclusively, to evaluate its reactivity towards the substrate. Moreover, the rate of 5-HT oxidation decreases with an increase in Cu–Aβ concentration from 0.74 mM to 1.38 mM (Fig. S9 and Table S1†). Note that the Cu–Aβ concentration dependence on substrate oxidation is consistent with the higher rate of dimerization of Cu(ii)–OOH to produce bis-μ-oxo dicopper(iii), as observed in EPR spectroscopy (Fig. 3B). These results unambiguously support that Cu(ii)–OOH is the active oxidant responsible for the oxidation of 5-HT. This is in contrast to the report by Ming *et al.*, in which they have proposed a side-on μ-peroxo dicopper(ii) intermediate as the active oxidant for Cu–Aβ for substrate oxidation in the presence of H_2_O_2_.^34,36^ Our conclusion agrees very well with the mechanism of enzymes like DβM, where the Cu(ii)–OOH species or species originating from it are responsible for the chemical oxidation of amino acids and neurotransmitters.^60–62^

**Fig. 6:**
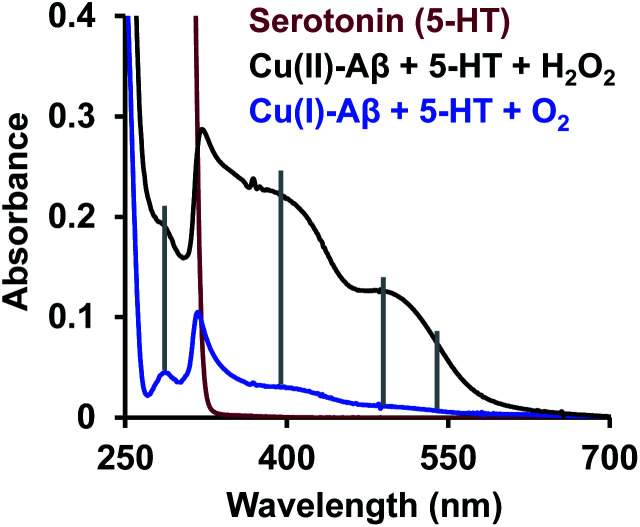
Absorption spectra of serotonin (5-HT), red; oxidation of 5-HT by H_2_O_2_ catalyzed by Cu(ii)–Aβ, black and by O_2_ catalyzed by Cu(i)–Aβ, blue; in 100 mM HEPES buffer at pH 7.

**Scheme 5:**
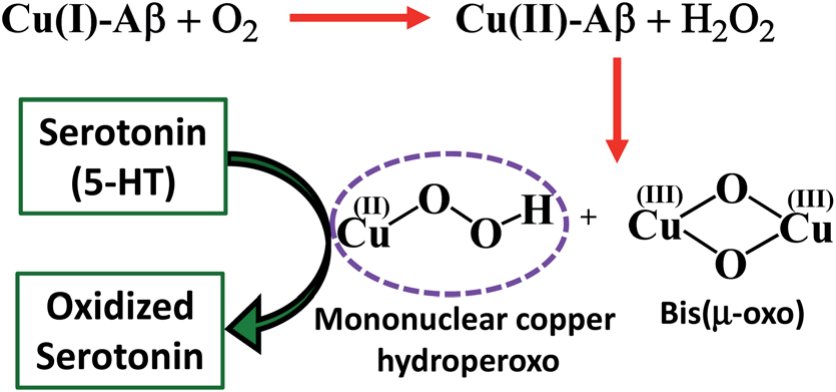
Serotonin oxidation catalyzed by a reactive oxidant.

Finally, reduced Cu–Aβ can react with O_2_, where the Cu gets oxidized and H_2_O_2_ is generated *via* disproportionation of the O_2_^−^ produced.^11^ The H_2_O_2_ generated from dissolved oxygen in blood can trigger the oxidation of serotonin. This is exactly the same case as indicated by the gradual appearance of the bands corresponding to the oxidized products of 5-HT when incubated with a solution of Cu–Aβ reduced with ascorbic acid in aerated buffer solutions (Fig. 6, blue), albeit the extent of oxidized HT produced is much less under these stoichiometric conditions. Ming *et al.* have previously observed a similar result as Cu–Aβ significantly accelerated the aerobic oxidation of the neurotransmitters.^34^Scheme 5 demonstrates the possible routes for the oxidation of serotonin.

## Conclusion

Cu–Aβ oxidizes the neurotransmitter serotonin (5-HT) in the presence of H_2_O_2_. The combined absorption, EPR and resonance Raman data indicate that Cu(ii)–Aβ reacts with H_2_O_2_ to produce bis(μ-oxo)dicopper and mononuclear copper hydroperoxo intermediates. This is the first experimental characterization of the active oxidants originating from Cu bound Aβ that can oxidize neurotransmitters like serotonin (5-HT) and generate neurotoxins like tryptamine-4,5-dione, which are also observed in an AD brain. Cu(ii)–Aβ can be reduced by physiologically relevant reductants like ascorbic acid and this reduced Cu center can generate H_2_O_2_ by reacting with dissolved oxygen, which can then oxidize 5-HT catalyzed by the Cu(ii)–Aβ produced. Both these pathways are accessible under physiological conditions and may account for the abnormal neurotransmission, a key pathological feature of AD.

## Conflicts of interest

There are no conflicts to declare.

## Supplementary Material

SC-012-D0SC06258H-s001
