# Supplementary material for: Intermediates involved in serotonin oxidation catalyzed by Cu bound Aβ peptides
Source: Chem Sci. 2020 Dec 22;12(5):1924–9. doi: 10.1039/d0sc06258h (PMC8179298; doi:10.1039/d0sc06258h)
Supplement: SC-012-D0SC06258H-s001 [file SC-012-D0SC06258H-s001.pdf]

Electronic Supplementary Material (ESI) for Chemical Science.
This journal is © The Royal Society of Chemistry 2020

Supporting Information

Intermediates Involved in Serotonin Oxidation Catalyzed by Cu bound Aβ Peptides

Arnab Kumar Nath, Arnab Ghatak, Abhishek Dey*, Somdatta Ghosh Dey*

Contents **Page Number**

**Materials and Methods**	**3-6**
**Figure S1**	**6**
**Figure S2**	**7**
**Figure S3**	**8**
**Figure S4**	**9**
**Figure S5**	**10**
**Figure S6**	**10**
**Figure S7**	**11**
**Figure S8**	**11**
**Figure S9**	**12**
**Scheme 1**	**12**
**Table 1**	**13**
**References**	**13**

Materials and Methods

All reagents were of the highest grade commercially available and were used without further purification. Both Aβ peptides of 1-16 and 1-40 chain length are used for UV-Vis experiments and kinetic assay experiments for serotonin oxidation. HPLC analysis for serotonin oxidation, EPR and resonance Raman experiments for the reaction of Cu-Aβ + H_2O_2 is performed with Aβ peptide of chain length 1-16. All peptides were purchased from Ontores, China with >95% purity. Copper sulfate ($CuSO_4$), Serotonin and HEPES buffer were purchased from Sigma. Sulphuric acid and NaOH were purchased from Merck. D_2O was purchased from Cambridge Isotope Laboratory.

All of the pH 7 peptide stock solutions were made in 100 mM N-(hydroxyethyl)piperazine-N'-2-ethanesulfonic acid (HEPES) buffer. Aβ stock solution of 1 mM strength was prepared by dissolving in 100 mM pH 7 HEPES buffer. In all the experiments Aβ(1-16) peptide is used. The Cu(II)-Aβ complex was prepared by incubating 0.8 equivalent of $CuSO_4$ with 1 equivalent of Aβ for ~1 h. The pH was maintained at 7 by adding H_2SO_4 and 1M NaOH. Strength of serotonin was 7.4 mM (10 eq) and was prepared by dissolving it in 100 mM HEPES buffer. H_2O_2 is prepared by diluting the 10 M stock solution to 1 M by Millipore water and aliquots were added to adjust the final concentration to the desired value (50-1000 eq.).

<u>Physical Methods</u>

Absorption spectra

Absorption spectra were recorded by adding H_2O_2 into ~300 μL of the Cu-Aβ complex solution in a cuvette of 1 cm path length. Concentration of Cu-Aβ were 0.16 mM, 0.74 mM and 1.38 mM and that of H_2O_2 were 100 mM, 1 M and 10 M respectively. Concentration of H_2O_2 in the final solution were 1.6 mM (0.16 mM Cu-Aβ + 10 eqv

H_2O_2), 7.4 mM (0.74 mM Cu-Aβ + 10 eqv H_2O_2), 37 mM (0.74 mM Cu-Aβ + 50 eqv H_2O_2), 69 mM (1.38 mM Cu-Aβ + 50 eqv H_2O_2). Absorption spectra were obtained by a UV-Vis diode array spectrophotometer (Agilent 8453).

Serotonin Oxidation

Absorption spectra and kinetics of serotonin oxidation was monitored in a UV-vis diode array spectrophotometer (Agilent 8453). The concentration of serotonin solution was 7.4 mM and 13.8 mM respectively. Strength of Cu-Aβ solutions were 0.16 mM, 0.74 and 1.38 mM and strength of H_2O_2 were 100 mM, 500 mM and 10 M respectively.

Kinetics experiments for serotonin oxidation for Cu-Aβ(1-16) (0.74 mM) + 50 eqv H_2O_2 were performed in the presence of 50 eqv serotonin Concentrations of Cu-Aβ, H_2O_2 and 5-HT in the final solution were 0.055 mM, 2.77 mM and 2.77 mM, respectively. Similarly serotonin oxidation for Cu-Aβ(1-16) (1.38 mM) + 50 eqv H_2O_2 in the presence of 50 eqv serotonin were also performed. Concentrations of Cu-Aβ, H_2O_2 and 5-HT in the final solution were 0.10 mM, 5.0 mM and 5.0 mM, respectively.

Kinetics experiments for serotonin oxidation for Cu-Aβ(1-16) (0.74 mM) + 1000 eqv H_2O_2 were performed by adding 50 eqv serotonin. Concentrations of Cu-Aβ, H_2O_2 and 5-HT in the final solution are 0.055 mM, 55.5 mM and 2.77 mM, respectively.

Comparison of serotonin oxidation for Cu-Aβ(1-16) and Cu-Aβ(1-40) was done by following the kinetics at 392 nm keeping Cu-Aβ : H_2O_2 :5-HT (1:50:50) and final concentrations 0.012 mM, 0.60 mM and 0.60 mM respectively.

EPR

EPR samples were 0.74 mM and 1.38 mM in concentration and 100 μL in volume and were run at 77 K in a liquid nitrogen finger dewar. EPR spectra were obtained by a JEOL FA200 spectrophotometer with the

following parameters: modulation width, 14 gauss; amplitude, 30; time constant, 300 ms; power, 1 mW; frequency, 9.14 GHz.

Resonance Raman

RR data were obtained using a Trivista 555 spectrograph (Princeton Instruments) using 415.4 nm excitation from a Kr^+ laser (Coherent, Sabre Innova SBRC-DBW-K). The power on the samples was ~10 mW. The rR samples were 1.38 mM in concentration and were prepared by adding 50 and 1000 equivalents of H_2O_2 to Cu-Aβ(1-16) and freezing the sample in liquid nitrogen at 77 K. For isotope labelling Cu-Aβ(1-16) is prepared in HEPES buffer (H_2O and D_2O) and then same protocols as that of H_2O are followed.

HPLC

Oxidation products of 5-HT by Cu–Aβ complexes were separated by reversed-phase HPLC (RP-HPLC) using a Waters 1525 Separation Module coupled to a diode-array detector (Waters 2487). A SymmetryR C18 reversed-phase column (250 mm X 4.6 mm internal diameter, 5 μm particle size) (SHIMADZU CORP.) was used to separate 5-HT from its oxidation products using the linear gradient method. The mobile phase consisted of eluant A (1 % acetic acid and 1 mM ammonium acetate in water) and eluant B (100 % acetonitrile). The gradients applied were 0–10 min, 98–85 % eluant A; 10–12 min, 85–50 % eluant A; 12–14 min, 50 % eluant A. The flow rate was 0.8 mL/min. For the HPLC assay, 7.4 mM 5-HT was incubated with Cu-Aβ (0.74 mM) + H_2O_2 (1 M for 50 eqv. and 10 M for 1000 eqv.) aliquot in 100 mM HEPES, pH 8 with a conc. ratio of Cu-Aβ, H_2O_2 and serotonin to be 1: 50/1000 : 50. At specific time intervals, the oxidation products were analysed by injecting 25 μL of the reaction mixture into the column. Serotonin and its oxidation products were analysed with a UV-online detector set at 260 nm.

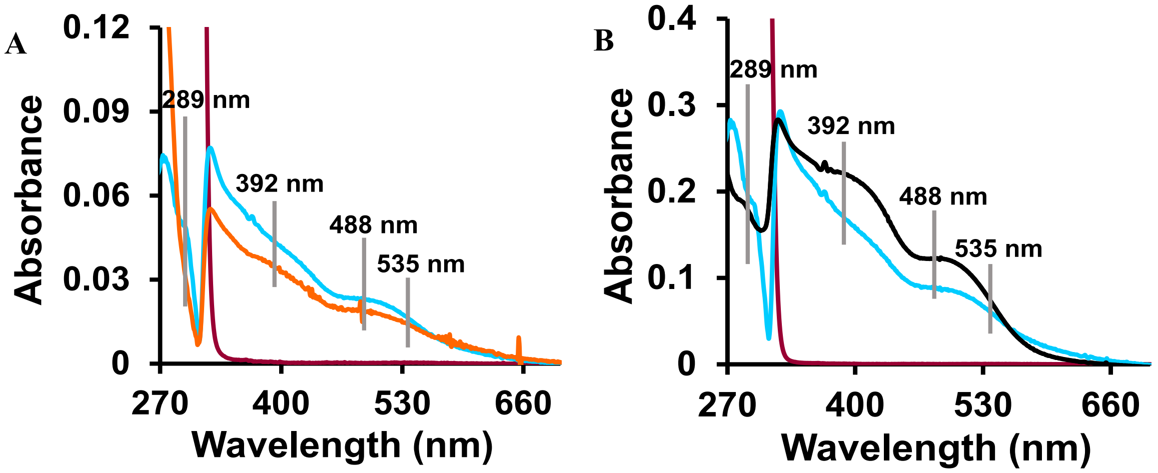

Figure S1. A) Absorption spectrum of serotonin (5-HT), red; for the reaction 0.012 mM Cu-Aβ(1-16) + 0.60 mM H_2O_2 + 0.60 mM 5-HT (1:50:50), cyan and 0.012 mM Cu-Aβ(1-40) + 0.60 mM H_2O_2 + 0.60 mM 5-HT (1:50:50), orange;

B) Absorption spectrum of serotonin (5-HT), red; absorption spectrum for the reaction 0.055 mM Cu-Aβ(1-16) + 2.77 mM H_2O_2 + 2.77 mM 5-HT (1:50:50), black and 0.012 mM Cu-Aβ(1-16) + 0.60 mM H_2O_2 + 0.60 mM 5-HT (1:50:50) scaled, cyan; in 100 mM HEPES buffer at pH 7.

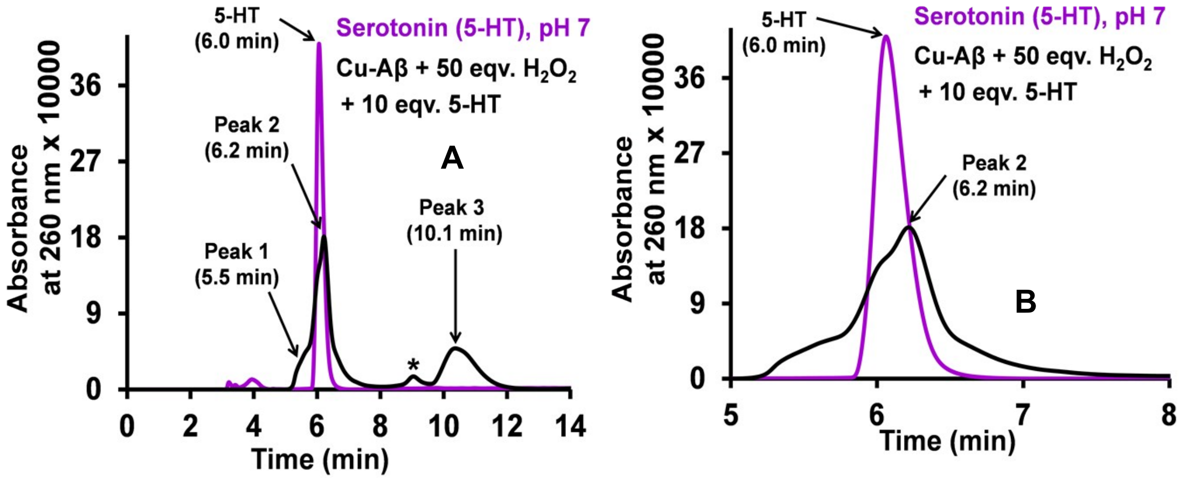

Figure S2: Separation of the 5-HT and its products by RP-HPLC after its incubation with Cu–Aβ + 50 eqv. H_2O_2; 5-HT without reaction mixture purple, separated reaction mixture at 5.5 min, 6.2 min and 10.1 min after incubation resulting in major products (peak 1, peak 2 and peak 3), black A) full range, B) elution time from 5 min to 8 min; in 100 mM HEPES buffer at pH 7. The peaks with star marking (*) is the minor product which is other oxidized products of serotonin.

HPLC shows that serotonin in the presence of Cu-Aβ and H_2O_2 oxidizes to 5-Hydroxy-3-ethylamino-2-oxindole (5-HEO) (peak 1) and tryptamine-4,5-dione (peak 2). In the chromatogram, peak 3 represents 3,3'-bis(2-aminoethyl)-5-hydroxy-[3,7'-bi-1H-indolel]-2,4',5'(3H)-trione which is the dimerized form of the two oxidized products, which correspond to peak 1 and peak 2 and possibly forms through aerial oxidation (Scheme S1).[1, 2]

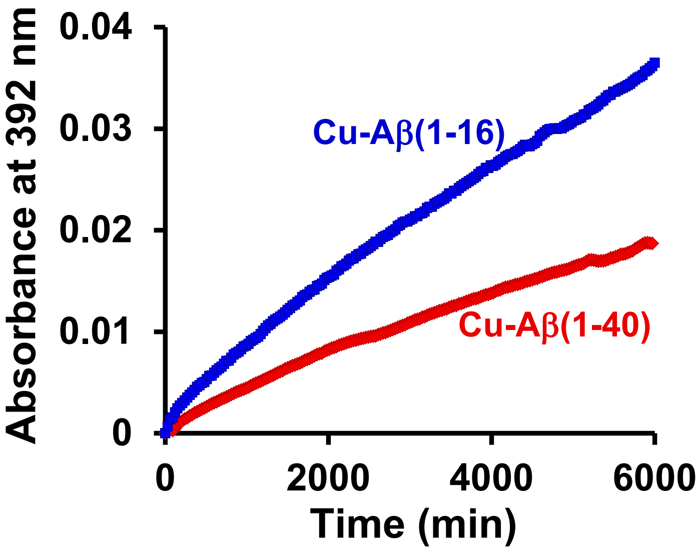

Figure S3. Kinetics of serotonin oxidation monitored at 392 nm for the reaction 0.012 mM Cu-Aβ(1-16) + 0.60 mM H_2O_2 + 0.60 mM 5-HT (1:50:50), blue and 0.012 mM Cu-Aβ(1-40) + 0.60 mM H_2O_2 + 0.60 mM 5-HT (1:50:50), red; in 100 mM HEPES buffer at pH 7.

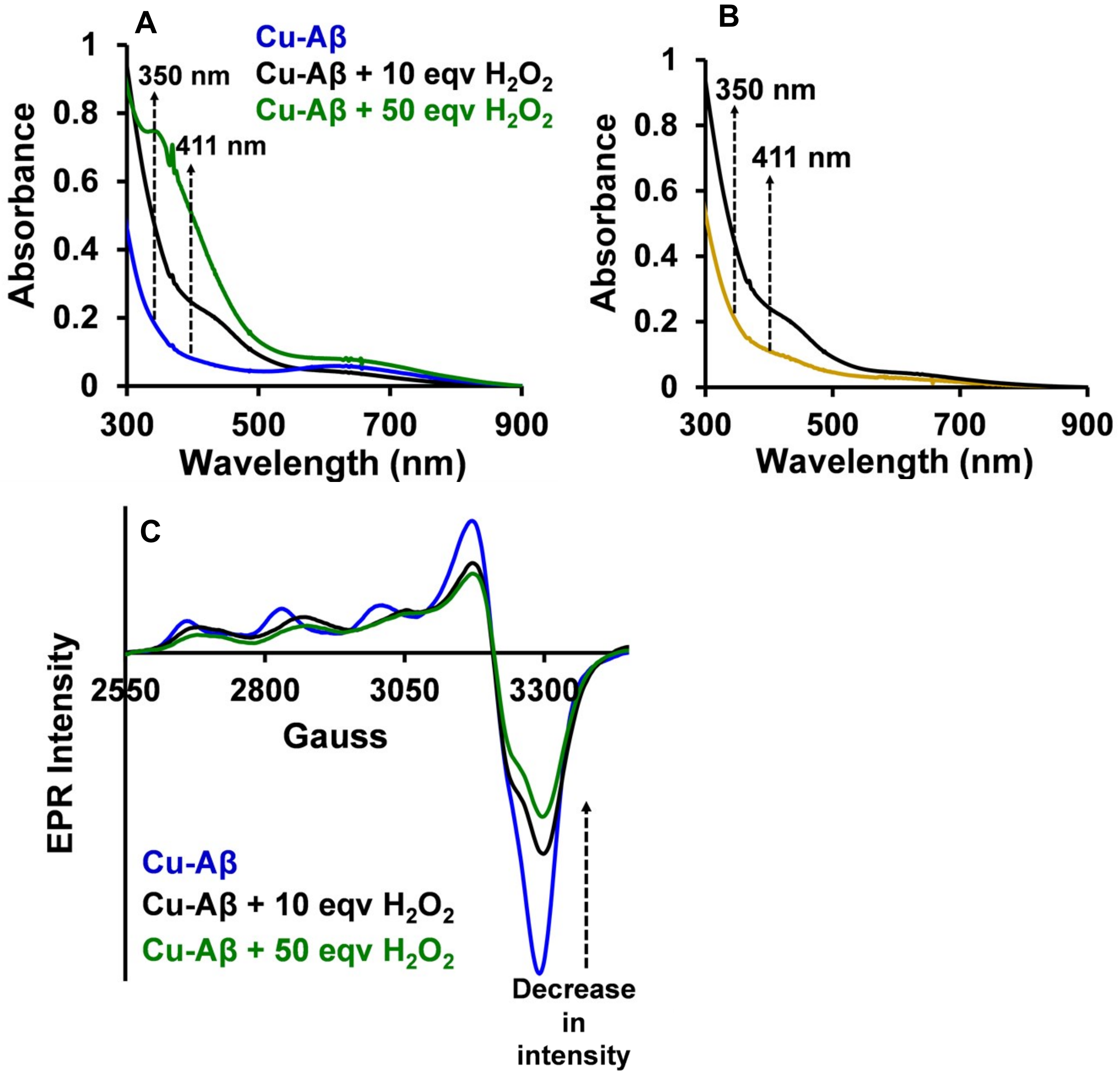

Figure S4. A) Absorption spectrum of Cu-Aβ, blue; for the reaction of 0.74 mM Cu-Aβ(1-16) + 7.4 mM H_2O_2 (1:10) after 2 hours, black and 0.74 mM Cu-Aβ(1-16) + 37 mM H_2O_2 (1:50) after 2 hours, green; in 100 mM HEPES buffer at pH 7.

B) Absorption spectrum for the reaction of 0.74 mM Cu-Aβ(1-16) + 7.4 mM H_2O_2 (1:10), black and 0.16 mM Cu-Aβ(1-16) + 1.6 mM H_2O_2 (1:10), yellow; in 100 mM HEPES buffer at pH 7.

C) EPR spectra of Cu-Aβ, blue; for the reaction of 0.74 mM Cu-Aβ(1-16) + 7.4 mM H_2O_2 (1:10) after 2 hours, black and 0.74 mM Cu-Aβ(1-16) + 37 mM H_2O_2 (1:50) after 2 hours, green, at 77 K; The arrows indicate the direction of the spectral changes. All data are collected in 100 mM HEPES buffer at pH 7, at 77 K.

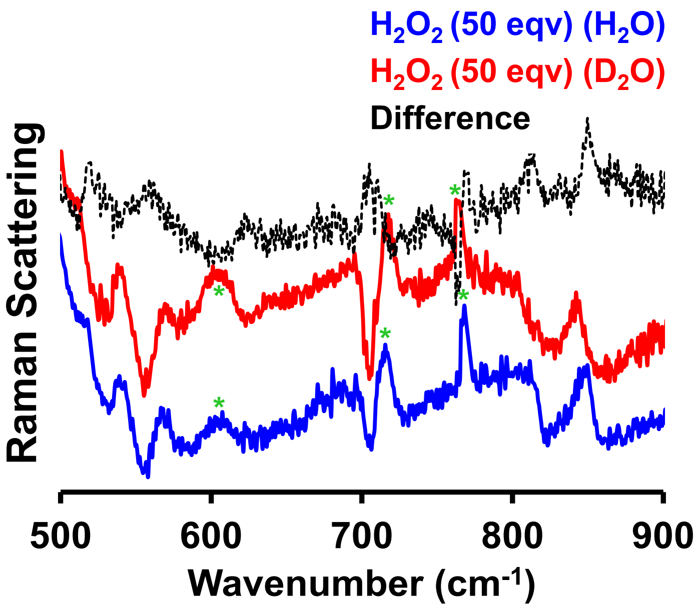

Figure S5: The extended spectra of Cu-Aβ + 50 eq. H_2O_2 (blue) and Cu-Aβ + 50 eq. D_2O_2 (red) and difference (dashed black). The peaks arising from glass tube and buffer are indicated with *.

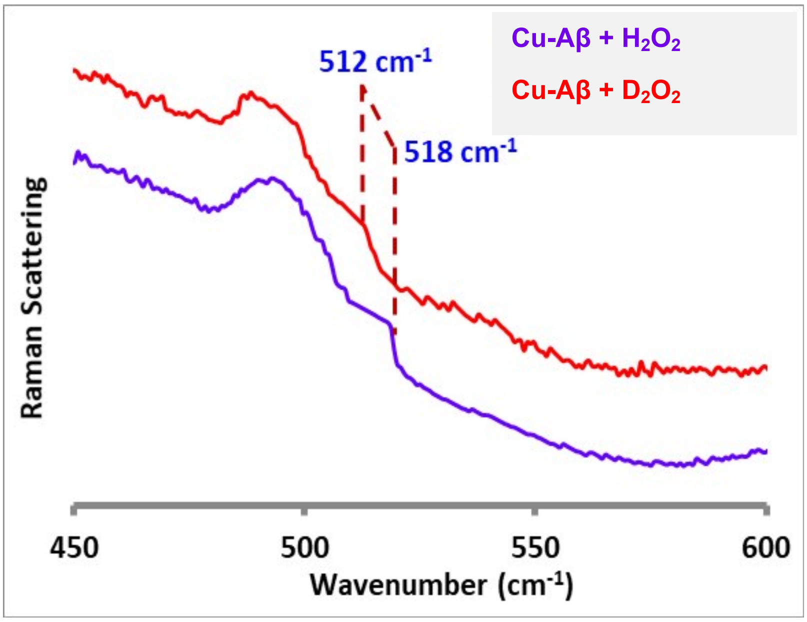

Figure S6: Cu-O vibration in Cu-Aβ + 1000 eq. H_2O_2 (purple) and Cu-Aβ + 1000 eq. D_2O_2 (red)

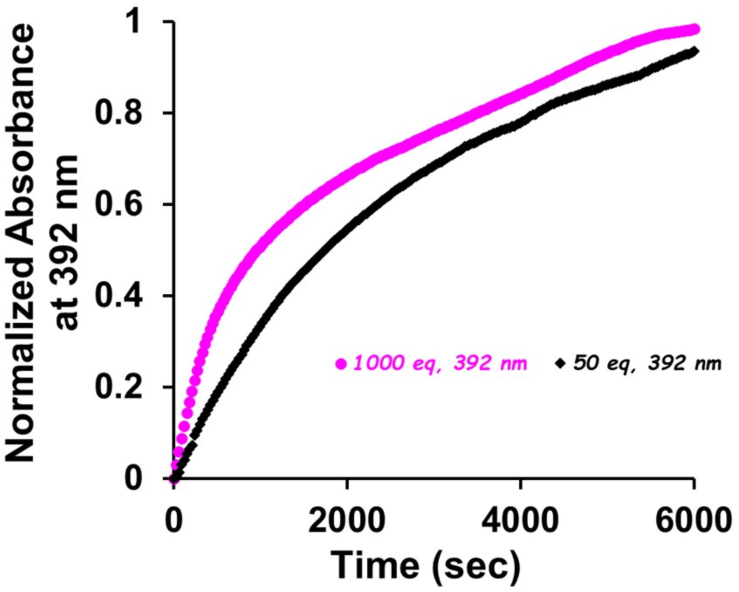

Figure S7: Kinetics of 5-HT oxidation catalysed by Cu-Aβ using 1000 eq. H_2O_2 (purple) and 50 eq. H_2O_2 (black).

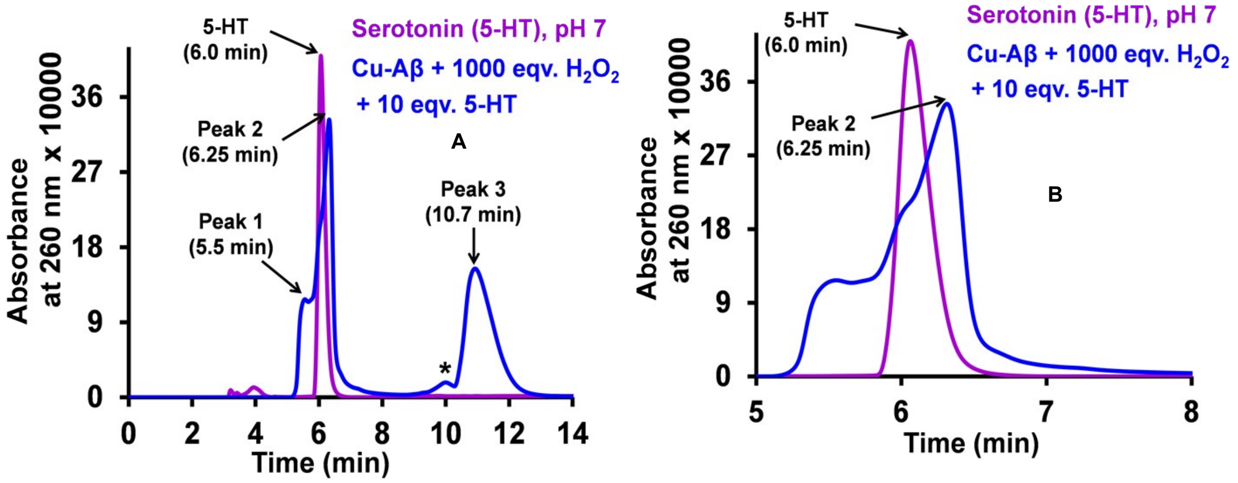

Figure S8: Separation of the 5-HT and its products by RP-HPLC after its incubation with Cu–Aβ + 1000 eqv. H_2O_2; 5-HT without reaction mixture purple, separated reaction mixture at 5.5 min, 6.25 min and 10.7 min after incubation resulting in major products (peak 1, peak 2 and peak 3), black A) full range, B) elution time from 5 min to 8 min; in 100 mM HEPES buffer at pH 7. The peaks with star marking (*) is the minor product which is other oxidized products of serotonin.

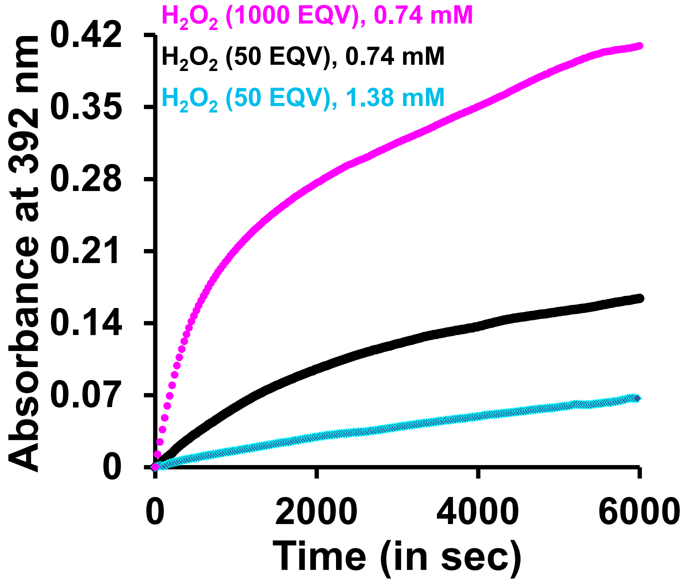

Figure S9: Kinetics of 5-HT oxidation catalysed by Cu-Aβ using Cu-Aβ (0.74 mM) + 1000 eq. H_2O_2 (purple), Cu-Aβ (0.74 mM) + 50 eq. H_2O_2 (black) and Cu-Aβ (1.38 mM) + 50 eq. H_2O_2 (cyan).

Scheme S1: Serotonin oxidation by Cu-Aβ with H_2O_2 (50 and 1000 H_2O_2) at pH 7

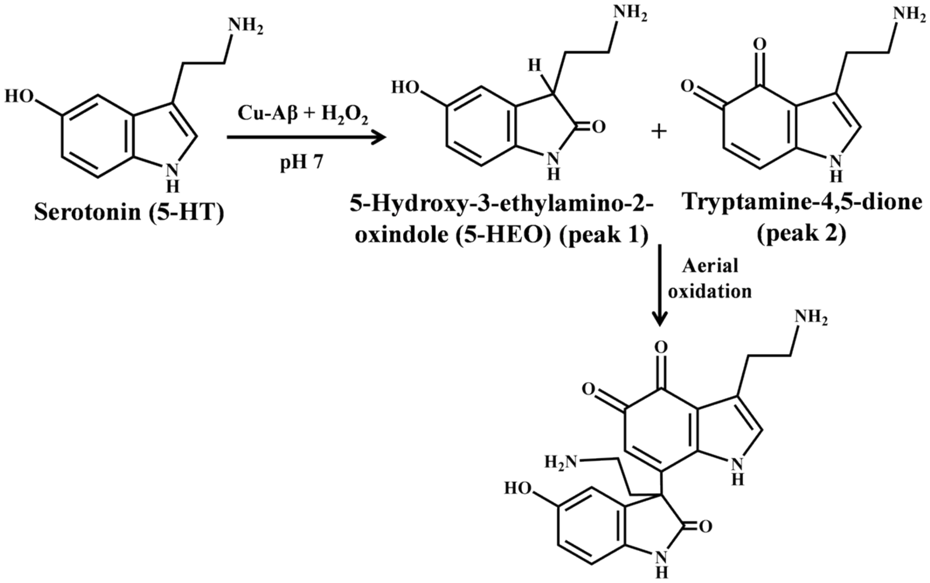

Table S1. Rate of serotonin oxidation for different Cu-Aβ + H_2O_2 conditions.

**Cu-Aβ + H_2O_2 for 10 eq 5-HT**	**Rate const. (Sec^{-1}) × 10^{-4}**
**Cu-Aβ (0.74 mM) + 1000 eq H_2O_2 at 15 min**	**6.2 ± 0.1**
**Cu-Aβ (0.74 mM) + 50 eq H_2O_2 at 100 min**	**4.0 ± 0.2**
**Cu-Aβ (1.38 mM) + 50 eq H_2O_2 at 100 min**	**2.5 ± 0.2**

References

1. M. Z. Wrona, Z. Yang, M. McAdams, S. O'Connor-Coates and G. Dryhurst, *Journal of Neurochemistry*, 1995, **64**, 1390-1400.
2. V. F. Ximenes, G. J. Maghzal, R. Turner, Y. Kato, C. C. Winterbourn and A. J. Kettle, *The Biochemical journal*, 2009, **425**, 285-293.
